# Community‐driven demand creation for the use of routine viral load testing: a model to scale up routine viral load testing

**DOI:** 10.1002/jia2.25009

**Published:** 2017-11-12

**Authors:** Bactrin M Killingo, Trisa B Taro, Wame N Mosime

**Affiliations:** ^1^ International Treatment Preparedness Coalition Nairobi Kenya; ^2^ International Treatment Preparedness Coalition New York NY USA; ^3^ International Treatment Preparedness Coalition Gaborone Botswana

**Keywords:** HIV treatment, routine viral load testing, community‐led advocacy, demand creation

## Abstract

**Introduction:**

HIV treatment outcomes are dependent on the use of viral load measurement. Despite global and national guidelines recommending the use of routine viral load testing, these policies alone have not translated into widespread implementation or sufficiently increased access for people living with HIV (PLHIV). Civil society and communities of PLHIV recognize the need to close this gap and to enable the scale up of routine viral load testing.

**Methods:**

The International Treatment Preparedness Coalition (ITPC) developed an approach to community‐led demand creation for the use of routine viral load testing. Using this Community Demand Creation Model, implementers follow a step‐wise process to capacitate and empower communities to address their most pressing needs. This includes utlizing a specific toolkit that includes conducting a baseline assessment, developing a treatment education toolkit, organizing mobilization workshops for knowledge building, provision of small grants to support advocacy work and conducting benchmark evaluations.

**Results and Discussion:**

The Community Demand Creation Model to increase demand for routine viral load testing services by PLHIV has been delivered in diverse contexts including in the sub‐Saharan African, Asian, Latin American and the Caribbean regions. Between December 2015 and December 2016, ITPC trained more than 240 PLHIV activists, and disbursed US$90,000 to network partners in support of their national advocacy work. The latter efforts informed a regional, community‐driven campaign calling for domestic investment in the expeditious implementation of national viral load testing guidelines.

**Conclusions:**

HIV treatment education and community mobilization are critical components of demand creation for access to optimal HIV treatment, especially for the use of routine viral load testing. ITPC's Community Demand Creation Model offers a novel approach to achieving this goal.

## Introduction

HIV treatment outcomes among people living with HIV (PLHIV) are dependent on monitoring the response to antiretroviral therapy (ART). The use of routine viral load testing (RVLT) to monitor this response is the gold standard, and has been recommended by the World Health Organization (WHO) in its treatment guidelines since 2013 1, 2. The UNAIDS 90‐90‐90 goals, of which the third 90 target aims for achieving viral suppression among 90% of PLHIV on ART, make the use of RVLT more relevant than ever. Many governments have adopted the WHO guidelines and updated their national HIV treatment guidelines to include RVLT for all PLHIV on ART. However, these policies alone have not translated into widespread implementation and consequently this has led to insufficient access for PLHIV 3, 4, 5. Civil society groups and communities of PLHIV have recognized the need to close this critical gap between policy and implementation. This has motivated such groups to take responsibility for creatinge demand for RVLT, and to holding governments and donors accountable for providing RVLT at scale 6.

Creating demand for any service – including RVLT – is complex. It centers around the education and mobilziation of recipients of care. It is only when affected communities are knowledgeable about their HIV treatment, including the value of viral load monitoring, that they become empowered to advocate for its availability. It is also through having such knowledge that they can confidently engage in policymaking and program implementation at national, regional, and global levels.

In this report, we present the model and methodology we developed for supporting the development of community‐led demand creation for the use of RVLT and discuss key outcomes from work done using this model.

## Methods

**Figure 1:**
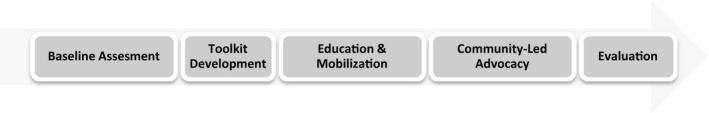
ITPC's Community Demand Creation Model.

The International Treatment Preparedness Coalition (ITPC) is a worldwide coalition of people living with HIV and community advocates working to achieve universal access to optimal HIV treatment of those in need. Formed in 2003 by a group of 125 HIV activists from 65 countries at a meeting in Cape Town, South Africa, ITPC actively advocates for treatment access in eight regions across the globe. ITPC's work over the last decade has focused heavily on treatment education and community mobilization to fuel demand creation for services along the HIV Continuum of Prevention, Care, and Treatment (CoPCT) Cascade 7. This model has been developed over the last 15 years through community consultative processes and lessons learned from best practices in community‐led programming (Figure 1).

In this model, implementers follow a step‐wise process to capacitate and empower communities to address their most pressing needs. Between December 2015 and December 2016, ITPC applied this model to focus specifically on increasing demand for RVLT services across the sub‐Saharan African, Asian, and Latin American and the Caribbean regions.

### Baseline assessment

As a first step, a baseline assessment is conducted to obtain a better contextual understanding of project needs (i.e. knowledge gaps to be addressed), to establish indicators and to serve as a baseline for project evaluation. This process usually entails a rapid or in‐depth contextual analysis, which guides subsequent steps of the ITPC Community Demand Creation Model. In December 2015, ITPC undertook a survey to assess levels of awareness and knowledge among PLHIV on ART in nine East and Southern Africa countries: Democratic Republic of Congo (DRC), Kenya, Lesotho, Malawi, Mozambique, South Africa, Swaziland, Uganda and Zimbabwe.. The survey demonstrated that the vast majority of PLHIV on ART surveyed had low levels of awareness and knowledge on the use of RVLT to monitor ART.

### Development of a community toolkit

**Figure 2:**
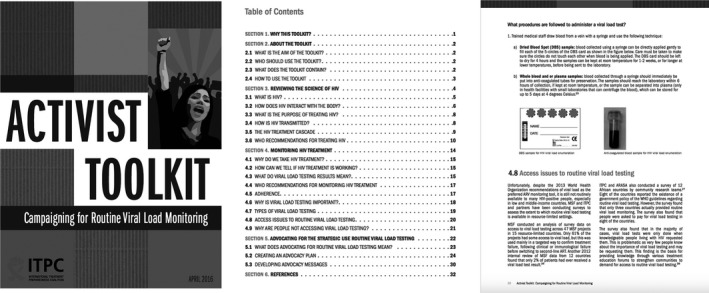
Activist Toolkit: Campaigning for Routine Viral Load Monitoring**.** cover (left), table of contents (center). *Excerpts provided here may appear different from official versions. Color was changed for purposes of publication.

The baseline assessment identifies several key focus areas used to guide the development of a treatment education toolkit. The toolkit consolidates an in‐depth literature review and contributions from technical experts in the field into a single resource utilized for training and knowledge building. In February 2016, ITPC launched its RVLT toolkit, guided by the outcomes of the baseline assessment conducted in East and Southern Africa. Entitled *Activist Toolkit: Campaigning for Routine Viral Load Monitoring*, the toolkit is a stand‐alone resource that communities can use to improve and expand their understanding of HIV treatment and HIV treatment monitoring using routine viral load testing. The content includes an overview of the science of HIV, HIV treatment, use of viral load testing to monitor HIV treatment and defining what advocacy for access to RVLT means (Figure 2). The toolkit also discusses strategies for tackling the most common advocacy issues, providing various case studies on demand creation for RVLT. Based on community needs and preferences, the tool is available in both print and soft copy format and in four languages: English, French, Portuguese, and Spanish 8.

### Education, skills building & mobilization

Following development and launch of the toolkit, in‐person treatment education and mobilization workshops are held to facilitate uptake and utilization of the tool. The toolkit provides content for workshops and facilitates the exchange required to increase knowledge and awareness around key issues. The aim of the workshops is two‐fold: (1) to provide community activists with the knowledge needed to demand RVLT scale‐up; and (2) to capacitate activists to apply this information in their contexts.

Between March and November 2016, ITPC held four regional workshops and nine national workshops. At these 3‐day workshops, participants included country representatives from various PLHIV networks, inclusive of representatives from key populations, women and youth groups.

The objectives of the workshops were: (1) to provide information about the importance of RVLT in the monitoring of HIV treatment; (2) to create national advocacy for addressing access to RVLT; and (3) to develop country awareness and advocacy plans regarding scale up of RVLT that feed into the *Be Healthy – Know Your Viral Load* campaign led by ITPC and its partners (described below).

ITPC facilitated the workshops alongside representatives from the relevant Ministry of Health and National Laboratory units. Pre‐ and post‐ tests, based on the content of the toolkit, helped assess changes in knowledge and awareness of participants – thereby helping to evaluate this component of the model.

Workshops culminated with the development of country‐specific advocacy plans to be implemented in the next step of the model. Through guided discussions during the workshop, participants identified and prioritized issues for targeted advocacy.

### Community‐led advocacy

To support the execution of the country awareness and advocacy plans developed during the workshops, small grants of US$10,000 are provided to national network partners. This step in the process is critical to ensuring that ideas are translated into action. Given the constrained funding environments that many national networks operate in, small grants provide dedicated funds for addressing the specific issues partners prioritize in their content.

Upon completion of each workshop, ITPC provided the small grants to each of the nine national workshop partners. Where there were multiple partners working in a country, a closed call for proposals was used to solicit the strongest partner. In most cases, country partners work in consortium with an internally nominated lead organization and a designated principle recipient of funds. Advocacy plans are conducted over the course of six to 12 months. Activities range from peer‐to‐peer education on RVLT to media engagements and policy dialogue on inclusion of RVLT into national ART guidelines and strategy to support its implementation and scale‐up.

In an effort to create synergy across national demand creation and advocacy work, ITPC and its partners have developed a community‐driven campaign titled *Be Healthy – Know Your Viral Load*. The campaign serves as the overarching umbrella within which all national‐level advocacy contribute, calling on governments to: (1) adopt the 2015 World Health Organization guidelines on the strategic use of routine viral load; (2) invest in the direct and strategic operationalization of guidelines that recommend the use of routine viral load testing; and (3) invest in the expeditious implementation of the RVLT guidelines at health facility level.

### Evaluation

The final stage of the ITPC Community Demand Creation Model is the conduct of an evaluation of the outcomes of the advocacy work to assess change achieved. Using the same methodology and indicators from the baseline assessment, a follow‐up survey is conducted to evaluate the country project as well as the overarching campaign. This process helps to strengthen future efforts for the application of the model.

## Results and Discussion

During 2016, ITPC conducted training for 242 PLHIV activists across Africa, Asia and Latin America and the Caribbean (Table 1). In all workshops, pre‐ and post‐test scores showed an increase in knowledge among participants. A single lead conducted all 13 workshops, alongside various support staff. Baseline knowledge among activists in‐country was extremely variable, with average pre‐test scores ranging from 57.6% at a regional workshop in Ethiopia, to 89.5% at a regional workshop in Thailand. This highlighted the need for further skill and knowledge building in countries that are lagging with respect to advocacy for RVLT.

**Table 1:** ITPC regional workshops on routine viral load testing held between march and november 2016

Type of workshop	Location	Number of participants	Countries represented	Average pre‐test score (%)	Average post‐test score (%)	Percent change in knowledge (based on pre‐ and post‐test)
Regional	Johannesburg, South Africa	29	9 ‐ South Africa, Swaziland, Lesotho, Mozambique, Zimbabwe, Malawi, Kenya, Uganda & DRC	Not available^a^	Not available^a^	Not Available^a^
	Addis Ababa, Ethiopia	20	5 – Kenya, Ethiopia, Rwanda, Burundi, Djibouti and Tanzania	57.6	84	+26.4
	Bangkok, Thailand	25	11 ‐ Nepal, Thailand, India, Cambodia, Myanmar, Bangladesh, Sri Lanka, Pakistan, Indonesia, China & Vietnam	89.5	93	+3.5
	Panama City, Panama	17	11 ‐ Nicaragua, Costa Rica, Panamá, Jamaica, Belize, Ecuador, Peru, El Salvador, Dominican Republic, Honduras and Guatemala	82.5	95	+12.5
National	Nairobi, Kenya	17	Kenya	89	97	+8
	Kampala, Uganda	15	Uganda	80	97	+17
	Kinshasa, Democratic Republic of Congo	19	Democratic Republic of Congo	83.3	90	+6.7
	Johannesburg, South Africa	17	South Africa	84.5	90	+5.5
	Mbabane, Swaziland	18	Swaziland	67.2	80	+12.8
	Maseru, Lesotho	17	Lesotho	76.5	94	+17.5
	Harare, Zimbabwe	16	Zimbabwe	87.7	91.1	+3.4
	Maputo, Mozambique	15	Mozambique	78	79.3	+1.3
	Lilongwe, Malawi	17	Malawi	79.7	93	+13.3

a Pre‐ and post‐tests were piloted at the first regional workshop in Johannesburg, South Africa and therefore formal data is not reportable.

By the end of 2016, the nine country partners had engaged 1631 individuals, including adolescents, women, and key populations, in treatment education activities. This engagement included active attendance and participation in officially organized sensitizations, workshops, and trainings using varying formats. They also carried out 168 advocacy actions involving 2041 individuals and distributed 7219 materials such as booklets, flyers and posters. As a result of these advocacy activities, four of the partners succeeded in securing new commitments from national decision makers to scale up viral load testing. For example, in Malawi, partners conducted a baseline situational analysis to identify the barriers, best practices and opportunities to scale up RVLT. The results of the survey informed the development of a stakeholder consortium through which groups of PLHIV, non‐governmental organizations (NGOs), and representatives from the Ministry of Health had regular meetings to discuss and plan for increased access to RVLT. As a result, the partner organizations were invited to join the Malawi Technical Working Group on ART organized by the Ministry of Health, to lead the group's work on viral load testing issues. This was an important first step in increasing civil society's voice and influence at the national policy level. In addition, the Malawi Community Health Services Unit committed to incorporating viral load testing information into their district‐wide training for all health service providers, another important achievement.

In Uganda, partner organizations held meetings to advocate with policy makers and key implementers, including the AIDS Control Programme in the Ministry of Health and with the National Coordinating Mechanism (CCM). As a result, partners secured the commitment for an increased number of viral load testing machines across the country, with support from the Ugandan Health System Strengthening Project, financed by the Global Fund. Furthermore, Ministry representatives committed to communicate in writing to all District Health Officers supporting the use of viral load testing for all PLHIV in Uganda. Such communications are critically important in disseminating information on national programmatic priorities. These initial powerful outcomes lay the groundwork for further advocacy.

National‐level advocacy for RVLT is conceptualized to also inform the broader global movement related to RVLT through facilitation of South‐to‐South knowledge sharing, consequently amplifying impact at the regional and global levels. However, coordination of national level advocacy in this way remains a challenge, not surprisingly due to varying national priorities. Throughout the 13 workshops, several key issues arose consistently across all countries; however, not surprisingly, the prioritization of these often varied based on the national context. For example, in Swaziland, participants felt that the lack of information on RVLT available to PLHIV was the most critical barrier to uptake. Whereas in the DRC, challenges with sample transportation called for advocacy around the use of dry blood spots (rather than plasma specimens) and difficulty in accessing treatment led to advocacy for differentiated service delivery models of care that promote task shifting to trained PLHIV groups to expand access to treatment more generally. Cross‐cutting initiatives, like the *Be Healthy – Know Your Viral Load* campaign, provide a meaningful approach to overcome these challenges and help in the harmonization of advocacy for RVLT across countries, allowing nationally specific advocacy plans to inform broader but regionally relevant overarching priorities.

ITPC's Community Demand Creation Model has strengths as well as limitations. The results presented here also highlight the ability of the model to be adapted across national and regional contexts. It can be applied at scale and in various resource‐limited settings based on both the needs of the community and the capability of the implementer partners within countries. However, all steps in the model (i.e. conducting an assessment, developing a tool, and conducting in‐person mobilization workshops) require significant investments in time and resources. Alternative methods of delivery, such as digital applications or webinars, could provide means for overcoming these limitations, helping expedite the process when operating under short timeframes and limited resources. Additionally, the adaptability of the model presents challenges with respect to evaluation, as universal indicators to assess the model's efficacy have yet to be developed. Civil society and community groups seeking to adopt this model should be encouraged to simultaneously establish monitoring and evaluation frameworks. As with any type of sub‐granting, selection of national partners must be approached in a systematic manner to ensure the organizational capacity exists to implement the proposed advocacy plans. Furthermore, and of important note, the capacity built within organizations through knowledge sharing, technical assistance, and financial support enable the establishment of sustainable capacity for advocacy beyond the life of the project. Thus, it is important that capacity and commitment are assessed before this type of investment in specific partner organizations is made.

## Conclusions

HIV treatment targets, including the UNAIDS 90‐90‐90 targets, will not be met without strengthening the capacity of PLHIV and activists to demand access to and use of RVLT. With expanded knowledge comes the ability to influence the entire viral load cascade. Thus, HIV treatment education and community mobilization are critical components of demand creation for access to optimal HIV treatment, including for the use of routine viral load testing. The work done using the ITPC Community Demand Creation Model serves as an example on how treatment education, coupled with small grants to civil society partner organizations, can create the desired outcomes for PLHIV and their communities.

## Competing interests

Authors have no competing interests to declare.

## Authors’ contributions

All authors contributed equally to this work. BK led the implementation of all aspects of the work on routine viral load testing presented here. WM provided technical oversight and implementation support. TT provided operational oversight and implementation support. TT wrote the main paper. All authors discussed the results and implications, and commented on the manuscript at all stages.
